# A systematic review of individual patient data meta-analyses on surgical interventions

**DOI:** 10.1186/2046-4053-2-52

**Published:** 2013-07-05

**Authors:** Gerjon Hannink, Hein G Gooszen, Cornelis JHM van Laarhoven, Maroeska M Rovers

**Affiliations:** 1 Department of Operating Rooms, Radboud University Nijmegen Medical Center, PO Box 9101, Nijmegen 6500HB, The Netherlands; 2 Department of Surgery, Radboud University Nijmegen Medical Center, Nijmegen, The Netherlands; 3 Department of Epidemiology, Biostatistics & HTA, Radboud University Nijmegen Medical Center, Nijmegen, The Netherlands

## Abstract

**Background:**

Compared to subgroup analyses in a single study or in a traditional meta-analysis, an individual patient data meta-analysis (IPDMA) offers important potential advantages. We studied how many IPDMAs report on surgical interventions, how many of those surgical IPDMAs perform subgroup analyses, and whether these subgroup analyses have changed decision-making in clinical practice.

**Methods:**

Surgical IPDMAs were identified using a comprehensive literature search. The last search was conducted on 24 April 2012. For each IPDMA included, we obtained information using a standardized data extraction form, and the quality of reporting was assessed. We also checked whether results were implemented in clinical guidelines.

**Results:**

Of all 583 identified IPDMAs, 22 (4%) reported on a surgical intervention. Eighteen (82%) of these IPDMAs presented subgroup analyses. Subgroups were mainly based on patient and disease characteristics. The median number of reported subgroup analyses was 3.5 (IQR 1.25-6.5). Statistical methods for subgroup analyses were mentioned in 11 (61%) surgical IPDMAs.

Eleven (61%) of the 18 IPDMAs performing subgroup analyses reported a significant overall effect estimate, whereas six (33%) reported a non-significant one. Of the IPDMAs that reported non-significant overall results, three IPDMAs (50%) reported significant results in one or more subgroup analyses. Results remained significant in one or more subgroups in eight of the IPDMAs (73%) that reported a significant overall result.

Eight (44%) of the 18 significant subgroups appeared to be implemented in clinical guidelines. The quality of reporting among surgical IPDMAs varied from low to high quality.

**Conclusion:**

Many of the surgical IPDMAs performed subgroup analyses, but overall treatment effects were more often emphasized than subgroup effects. Although, most surgical IPDMAs included in the present study have only recently been published, about half of the significant subgroups were already implemented in treatment guidelines.

## Background

Surgery has advanced spectacularly in the past 50 years, but many advances have not come from carefully planned research using valid study designs [1]. Research on surgical interventions is associated with several methodological and practical challenges of which few, if any, apply only to surgery. Surgical innovation is especially demanding because many of these challenges coincide [2]. Perhaps this situation leads many surgeons to view randomized controlled trials (RCTs), although theoretically advantageous, to be too difficult and impractical to undertake, and even worse, irrelevant to their practice because of concerns about generalizability [2,3].

The results of RCTs are usually implemented in practice by either treating or testing all patients in case of a ‘positive’ study or treating or testing no-one in case of a ‘negative’ study. Clinicians intuitively know that this approach is oversimplified because in reality some patients benefit more than average whereas others do not benefit. This may explain why around 50% of the RCTs perform subgroup analyses [4,5]. However, misleading claims about subgroup effects based on a single study are common [6].

Investigating subgroups is a highly relevant, but complex topic because of two interrelated concerns: failure to detect a relevant subgroup effect (false negative), and a misleading claim about a subgroup effect which in reality does not exist (false positive). Both of these problems can lead to suboptimal care for patients. Subgroup effects have been extensively and fiercely debated in the clinical, epidemiological, and statistical literature, especially in the context of single trials or traditional meta-analyses based on published summary results [7-11].

Individual patient data meta-analyses (IPDMAs) differ from traditional meta-analyses in that an IPD meta-analysis uses the ‘raw data’ of individual patients from included studies instead of the published summary results of studies in a traditional meta-analysis [12]. Compared to subgroup analyses in a single study or in a traditional meta-analysis, an IPDMA offers important potential advantages, such as: (1) increased possibilities to perform more complex statistical analyses that better match the underlying data; (2) more power compared to single studies and traditional meta-analyses; (3) higher validity of subgroup analyses by avoiding ecological bias and by taking the distribution of other patient characteristics into account; (4) improved flexibility and standardization of defining subgroups across studies; and (5) opportunities to examine the consistency of subgroup effects across studies [13-17].

In this paper we present a systematic overview of all IPDMAs on surgical interventions published. We studied the number and types of subgroup analyses performed, and whether these subgroups analyses influenced decision-making in clinical practice.

## Methods

### Search

A comprehensive literature search in PubMed, Embase, Web of Science, and the Cochrane Library was conducted to identify all IPDMAs of RCTs. The last search was conducted on 24 April 2012. Keywords used to develop our search strategy were ‘individual patient data’ and ‘meta-analysis’ (see Additional file 1 for detailed search strategy).

### Selection

In first instance, titles and abstracts were screened to identify eligible IPDMAs. Selection of potential eligible IPDMAs was restricted to IPD obtained from RCTs comparing surgical interventions. Patients had to be randomized over a surgical intervention in at least one treatment arm, and the surgical procedures had to be performed under general, spinal, epidural, or regional anesthesia. IPDMAs regarding drug-eluting medical devices and surgical trials in which a drug was the comparison were excluded.

Full text papers were retrieved when meta-analytic techniques for individual patient data of RCTs were used. IPDMAs using the same dataset or combination of datasets, studying/addressing different questions/subgroups were included. If obvious duplicate papers were available, the most elaborate paper was included.

### Data extraction and analysis

Data from all included surgical IPDMAs were extracted with respect to specific characteristics, that is, publication year, number of included trials and patients, domain, type of intervention, comparison, and outcome measured. Regarding the subgroups, number, type, justification, statistical methods, and results in relation to the overall effect estimate were studied.

We classified five types of subgroups, patient characteristics (for example, age or gender), disease characteristics (for example, severity or co-morbidity), household characteristics (for example, socioeconomic status or smoking), intervention characteristics (for example, type of intervention or dose), and methodological characteristics (for example, quality of included trials or trial effect). Justification for subgroups analyses was categorized as based on literature, clinical experience, biological mechanism, or no justification.

We also assessed the quality of reporting of all selected IPDMAs. IPDMAs on RCTs should be reported according to the Preferred Reporting Items for Systematic Reviews and Meta-Analyses (PRISMA) [18]. Since this guideline is not specific to IPDMAs, it has been suggested that some additional information should be reported, for instance why the IPDMA approach was initiated, whether there was a protocol for the IPDMA project, and whether a one-step or a two-step analysis was performed [12]. We judged the quality of reporting based on the 18 criteria suggested by Riley *et al.*[12]. Two independent reviewers (GH and MMR) selected eligible surgical IPDMAs and extracted data (duplicate independently). Any disagreements were resolved by consensus.

Finally, we reviewed available clinical guidelines for recommendations based on significant results of subgroup analyses from IPDMAs to determine the extent to which these results were implemented in clinical guidelines. We conducted a PubMed search for fields ‘patients’ (for example, carotid stenosis), ‘intervention’ (for example, carotid stenting) and ‘comparison’ (for example, endarterectomy), extracted from IPDMAs with significant subgroup analyses, and limited our search to ‘Practice Guideline’. We only included publications in English. We also searched the National Library of Guidelines (http://guidance.nice.org.uk/), and the National Guideline Clearinghouse (http://www.guidelines.gov/).

All steps in this review were carried out according to a pre-defined protocol (Additional file 2).

## Results

### Search

**Figure 1:**
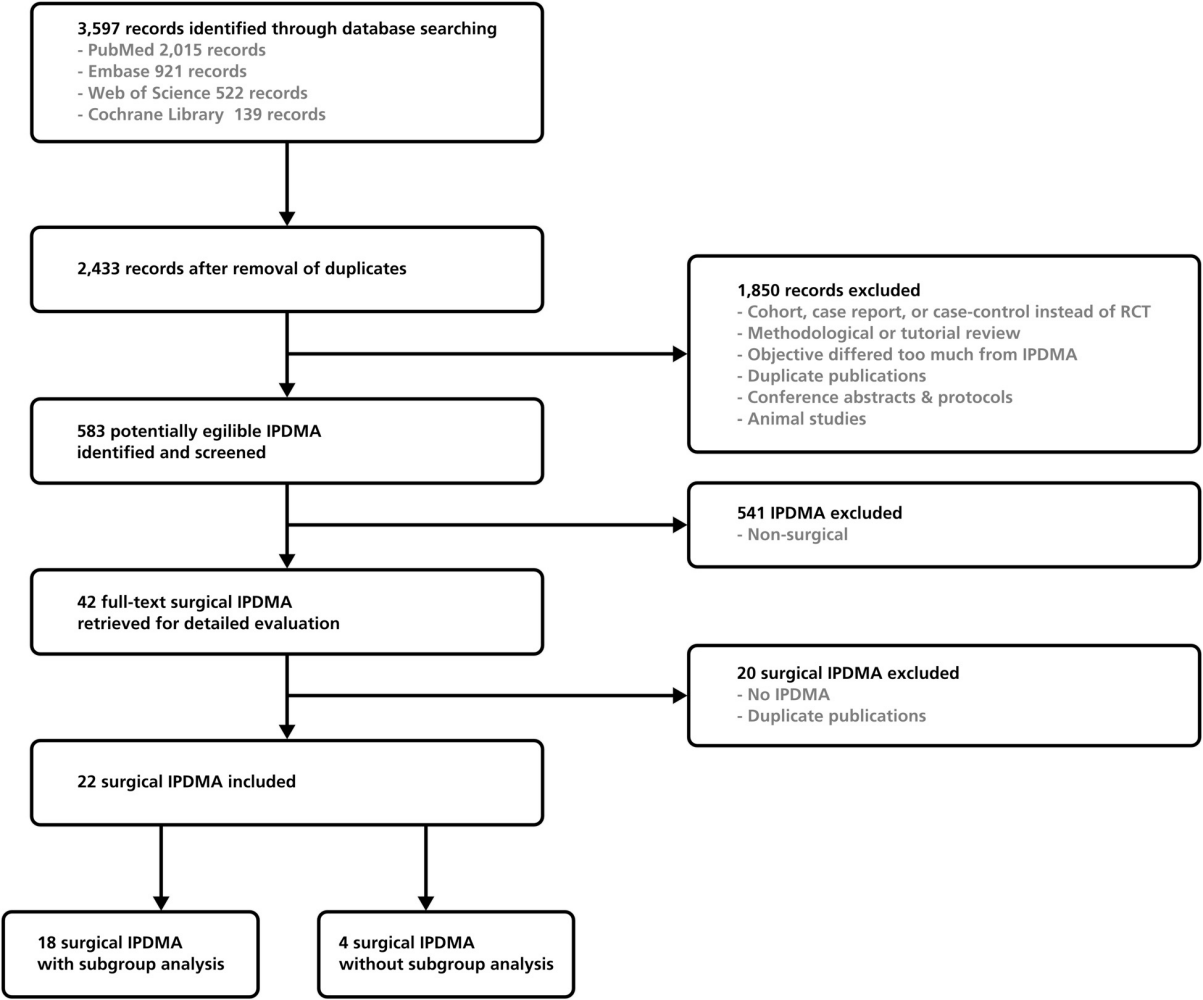
Flowchart of study selection process for IPDMA of surgical interventions.

In the search for IPDMAs, 3,597 potential eligible papers were identified. After studying the abstracts, 583 papers, published between 1991 and 2012, indeed reported an IPDMA. After detailed evaluation, 22 (4%) IPDMAs reported on a surgical intervention and met our inclusion criteria (Figure 1; Additional file 3).

Of the 22 surgical IPDMAs, 12 focused on cardiovascular interventions, three on inguinal hernia repair, three on gynecological interventions, two on orthopedic interventions, one on a gastroenterological intervention, and one on ventilation tubes for otitis media (Table 1). The surgical IPDMA papers were published between 2005 and 2012. Eighteen (82%) of the 22 surgical IPDMAs tried to identify subgroups of patients that benefit more or less from the surgical intervention.

**Table 1:** Characteristics of the 22 identified surgical IPDMAs

**Author and year**	**RCTs ( *n *)**	**Patients ( *n *)**	**Patients**	**Intervention**	**Comparison**	**Outcome**	**Subgroups ( *n *)**	**Overall effect estimate**	**Significant effect estimates in subgroups ( *n */ *N *)**
Jorgenson *et al.*, 2007 [19]	7	2,091	Women with cervical insufficiency	Cervical cerclage	Expectant management, no cerclage	*Primary*	Obstetric history, cervical length (2)	NS	0/2
						Pregnancy loss or neonatal death before discharge from hospital			
						*Secondary*			
						Preterm delivery and maternal morbidity			
Hlatky *et al.*, 2009 [20]	10	7,812	Patients with multivessel coronary disease	Coronary artery bypass graft	Percutaneous coronary intervention	All-cause mortality	Age, sex, diabetes, smoking, hypertension, hypercholesterolaemia, PVD, stability of symptoms, previous MI, heart failure, LV function, no. of diseased vessel, proximal LAD, balloon *vs.*stent (14)	NS	2/14
Daniels *et al.*, 2010 [21]	5	862	Patients with chronic pelvic pain	Laparoscopic uterosacral nerve ablation (LUNA)	No LUNA	Derived measure of worst pain level experienced	Presence of visual pathology, site of pain, age, parity (4)	NS	1/4
Burzotta *et al.*, 2009 [22]	11	2,686	Patients with ST-elevation myocardial infarction (STEMI)	Percutaneous coronary intervention with thrombectomy	Standard percutaneous coronary intervention	*Primary*	Manual *vs.* non-manual thrombectomy devices, diabetes, primary *vs.* rescue PCI, treated *vs.* non-treated with IIb/IIIa-inhibitors, ischemic time, infarct-related artery, pre-PCI TIMI flow (7)	S	1/7
						All-cause mortality			
						*Secondary*			
						Survival free from MI, TLR, or TVR, major adverse coronary events (MACE), death+MI			
Carotid Stenting Trialists’Collaboration, 2010 [23]	3	3,433	Patients with symptomatic carotid stenosis	Carotid stenting	Endarterectomy	*Primary*	Age, sex, diabetes, hypertension, SBP, hypercholesterolaemia, smoking, coronary heart disease, peripheral artery disease, most recent ipsilateral ischemic event, history of stroke, degree of ipsilateral ischemic stroke, contralateral severe carotid stenosis or occlusion, treatment within 14 days, patients recruited per center, center recruitment rate (16)	S	1/16
						Any stroke or death			
						*Secondary*			
						Disabling stroke or death, all-cause death, any stroke, myocardial infarction, severe local hematoma, severe wound infection			
Middleton *et al.*, 2010 [24]	17	2,814	Patients with heavy menstrual bleeding	Hysterectomy, endometrial destruction (1st & 2nd generation), levonorgestrel releasing intra-uterine system (MIRENA)	Endometrial destruction (1st & 2nd generation), levonorgestrel releasing intra-uterine system (MIRENA)	Dissatisfaction rates	Uterine cavity length, age, presence of fibroids/polyps, parity, baseline bleeding score (5)	S	1/5
Mercado *et al.*, 2005 [25]	4	3,051	Patients with multi-system coronary artery disease	Percutaneous coronary intervention with multiple stenting	Coronary artery bypass graft	*Primary*	Age, gender, diabetes, smoking, number of diseased vessels (5)	NS	0/5
						Composite of death, MI, or stroke at 1 year FU			
						*Secondary*			
						Death, composite of death or MI, repeat revascularization, composite of death, MI, stroke, and repeat revalscularization			
Boersma *et al.*, 2006 [26]	22	6,767	Patients with acute myocardial infarction	PCI	Fibrinolysis	All-cause mortality	Age, sex, diabetes, prior MI, MI location, heart rate, SBP, fibrinolytic agent, front-loaded tPA, site volume (11)	S	1/11
Timmer *et al.*, 2007 [27]	19	6,315	Patients with acute myocardial infarction	PCI	Fibrinolysis	Death, recurrent MI, death or recurrent MI, stroke	Diabetes (1)	S	0/1
de Boer *et al*., 2010 [28]	22	6,767	Patients with acute myocardial infarction	Primary PCI	Fibrinolysis	*Primary*	Age (1)	S	0/1
						All-cause mortality			
						*Secondary* reMI, stroke, composite of all-cause mortality or reMI, composite of all-cause mortality, reMI, or stroke			
de Boer *et al.*, 2011 [29]	22	6,767	Patients with acute myocardial infarction	Primary PCI	Fibrinolysis	All-cause mortality	High-risk patients (1)	S	0/1
Fox *et al.*, 2010 [30]	3	5,467	Patients with non-ST-elevation myocardial infarction	Routine invasive strategy	Selective invasive strategy	*Primary*	High-risk groups based on baseline characteristics (1)	S	1/1
						Composite of CV death or non-fatal MI			
						*Secondary*			
						All-cause death, non-fatal MI alone			
Damman *et al.*, 2012 [31]	3	5,467	Patients with non-ST-elevation myocardial infarction	Routine invasive strategy	Selective invasive strategy	*Primary*	Age (1)	S	1/1
						Composite of CV death or non-fatal MI, CV death, MI			
Damman *et al.*, 2012 [32]	3	5,467	Patients with non-ST-elevation myocardial infarction	Routine invasive strategy	Selective invasive strategy	All-cause mortality	Procedure-related MI, spontaneous MI (2)	S	1/2
Biau *et al.*, 2009 [33]	6	423	Patients with symptomatic unilateral anterior cruciate ligament injury	Reconstruction with patellar tendon autograft	Reconstruction with hamstring tendon autograft	*Primary*	Gender, age at surgery, trial effect (3)	S	2/3
						Positive pivot-shift test *Secondary*			
						Positive Lachman test			
Rovers *et al.*, 2005 [34]	7	1,234	Children with otitis media with effusion	Short-term ventilation tubes	Watchful waiting	Mean time spent with effusion, hearing, language development	Hearing level at baseline, history of acute otitis media, upper respiratory infections, attending day care, socioeconomic status, siblings, season, history of breastfeeding, parental smoking (9)	NS	2/9
Salerno *et al.*, 2007 [35]	4	305	Cirrhotic patients with refractory ascites	Transjugular intrahepatic portosystemic shunt (TIPS)	Paracentesis	*Primary*	NA	S	NA
						Death from any cause before LT			
						*Secondary*			
						Liver-related death			
Staples *et al.*, 2011 [36]	2	209	Patients with osteoporotic vertebral compression fractures	Vertebroplasty	Sham	Scores for pain and function	Onset of pain, pain scores at baseline (2)	NS	0/2
McCormack *et al.*, 2003 [37]	25	4,165	Patients with clinical diagnosis of groin hernia for whom surgical management was judged appropriate	Laparoscopic repair	Open repair	Duration of operation, ‘opposite’ method initiated, conversion, hematoma, seroma, wound/superficial infection, mesh/deep infection, port site hernia, vascular injury, visceral injury, length of hospital stay, time to return to usual activities, persisting pain, persisting numbness, hernia recurrence, known death within 30 days of surgery	NA	S	NA
				(Transabdominal preperitoneal repair (TAPP) or totally extraperitoneal repair (TEP))					
Scott *et al.*, 2002 [38]	11	3,347	Patients with clinical diagnosis of groin hernia for whom surgical management was judged appropriate	Mesh technique	Non-mesh technique	Duration of operation, ’opposite’ method initiated, conversion, hematoma, seroma, wound/superficial infection, serious complications, length of postoperative hospital stay, time to return to usual activities, persisting pain, persisting numbness, hernia recurrence, known death	NA	S	NA
EU Hernia Trialists Collaboration, 2002 [39]	35	6,901	Patients with clinical diagnosis of groin hernia for whom surgical management was judged appropriate	Laparoscopic repair, mesh methods	Open repair, non-mesh methods	Hernia recurrence, persisting pain	NA	S	NA
Gregson *et al.*, 2012 [40]	8	2,186	Patients with spontaneous supratentorial intracerebral hemorrhage	Surgery	Conservative treatment	Unfavorable outcome	Location of hematoma, time from event, age, Glascow Coma Score, volume of hematoma (5)	NA	4/5

**Figure 2:**
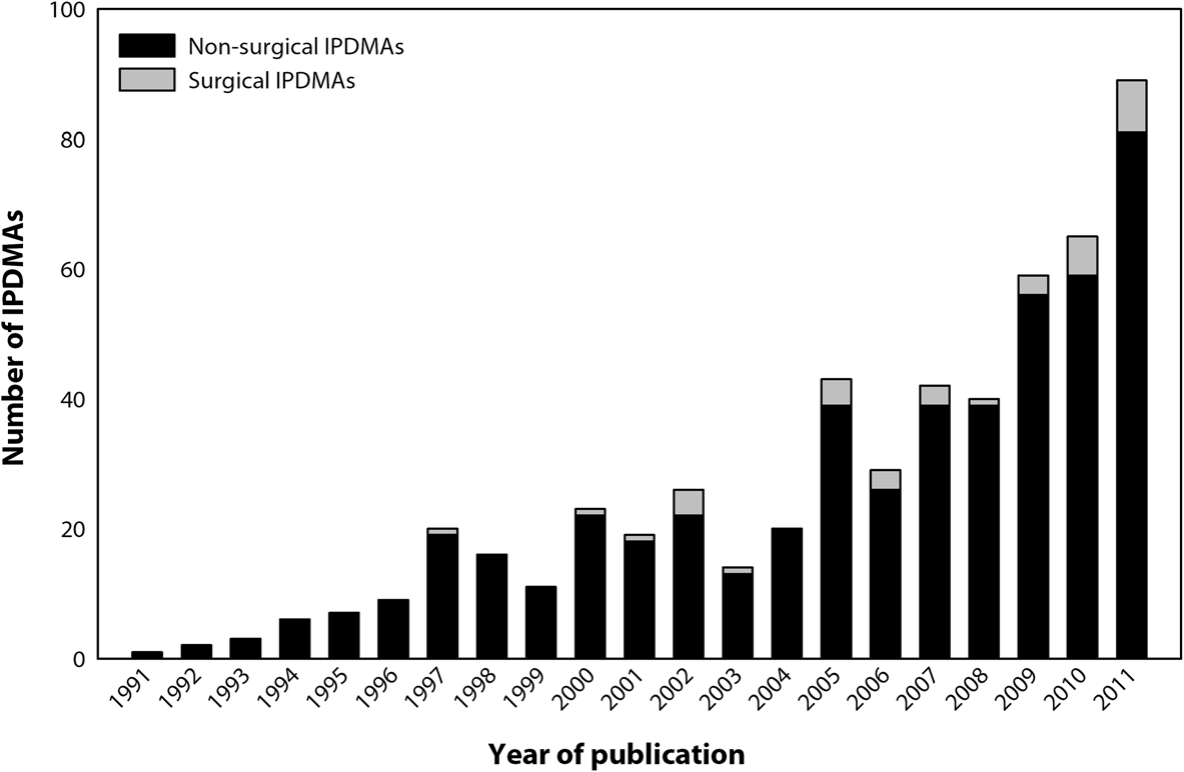
Number of applied IPDMA published up to April 2012,* as identified by a systematic review of PubMed, Embase, Web of Science, and the Cochrane Library. *Thirty-seven IPDMAs published in 2012 were identified up to 24 April 2012, when the review was conducted.

The remaining non-surgical IPDMAs predominantly focused on cancer, cardiovascular disease, and diabetes, and most assessed whether a treatment or intervention was effective, often in subgroups of patients. Before 2000 only a few IPDMAs were published, whereas a considerable rise in the number of published IPDMAs is seen between 2005 and 2012 (Figure 2). This growth is most likely the result of an increased awareness the potential advantages of IPDMAs, and the initiation of collaborations to specifically perform such studies.

### Summary of IPDMAs using IPD (or part of IPD) from the same trials

Of the 12 IPDMAs that focused on a cardiovascular intervention, four IPDMAs [26-29] used individual patient data from the same 22 trials (6,763 patients) evaluating the clinical effects of primary percutaneous coronary intervention *versus* in-hospital fibrinolysis. In three IPDMAs [30-32], comparing routine invasive strategies with selective invasive strategies in 5,467 patients with non-ST segment elevation acute coronary syndromes, data from the same three trials (FRISC II, ICTUS, and RITA-3) were used. In addition, of the three IPDMAs that focused on inguinal hernia repair one IPDMA [39] presented a combination of the data used in the other two IPDMAs [37,38].

### Number, justification, type, and methods of subgroups analyses in surgical IPDMAs

In 18 (82%) of the full set of surgical IPDMAs assessed, subgroup analyses were performed to examine whether certain patients benefit more from a specific treatment than others. The median number of subgroups reported in these IPDMAs was 3.5 (range, 1–16, IQR 5.25 (1.25-6.5)). In 12 (67%) of the 18 surgical IPDMAs that studied subgroups a justification for subgroup analyses was mentioned. Scientific literature was used for justification in these studies.

The types of subgroups studied varied. Fifteen (83%) IPDMAs studied patient characteristics, five (28%) studied household characteristics, 15 (83%) studied disease characteristics, and six (33%) studied intervention-related subgroups. Subgroups related to study or trial effects were studied in three (17%) IPDMAs. No IPDMAs studied subgroups related to the quality of the included trials, for example concealment of allocation, blinding, or completeness of follow-up.

Twelve (55%) IPDMAs stratified their analysis per trial before pooling the results (two-step analysis). A one-step analysis was performed in four (18%) IPDMAs. Statistical methods for subgroup analyses were mentioned in 11 (61%) of the 18 IPDMAs performing subgroup analyses. All IPDMAs that mentioned statistical methods for subgroup analysis used interaction tests.

Only five (28%) surgical IPDMAs mentioned the power of the subgroup analyses. Three IPDMAs reported that their studies were underpowered to detect subgroup effects, one IPDMA reported that their study was well powered to detect subgroups effects, however, did fail to show differences in subgroups, and one IPDMA mentioned differences in power between different subgroups, but not whether these were over- or underpowered.

Eleven (61%) of the 18 IPDMAs performing subgroup analyses reported a significant overall effect estimate, whereas six (33%) reported a non-significant one. One IPDMA (6%) did not report an overall effect estimate and only presented results of subgroup analyses [40].

Of the IPDMAs that reported non-significant overall results, three IPDMAs (50%) reported significant results in one or more subgroup analyses. Results remained significant in one or more subgroups in eight of the IPDMAs (73%) that reported a significant overall result.

Thirty-six (40%) of the total number of 90 subgroups analyses were performed on a non-significant or inconclusive overall effect estimate, whereas 49 (54%) were performed on a significant overall effect estimate. The remaining five (6%) subgroups originated from the one IPDMA that did not report an overall effect estimate and only presented results of subgroup analyses, four out of these five subgroups being significant [40]. Of the subgroup analyses performed on non-significant overall results, five (14%) became significant. Nine (18%) of those performed on IPDMAs with a significant overall result remained significant.

Eight (67%) of the 12 surgical IPDMAs with significant subgroups reported on what the implications of these significant results of their subgroup analyses were for clinical practice. Mainly, the importance of differentiating when evaluating the efficacy and safety of new medical and interventional treatments, and translating these findings in treatment recommendations were emphasized. Moreover, it was reported that the influence of certain subgroups had not been reported previously, that findings concurred with recent recommendations or guidelines, and that subgroups not *per se* needed to be an exclusion criterion for treatment. Eight (44%) of the 18 significant subgroups were implemented in clinical guidelines.

### Quality of reporting

**Figure 3:**
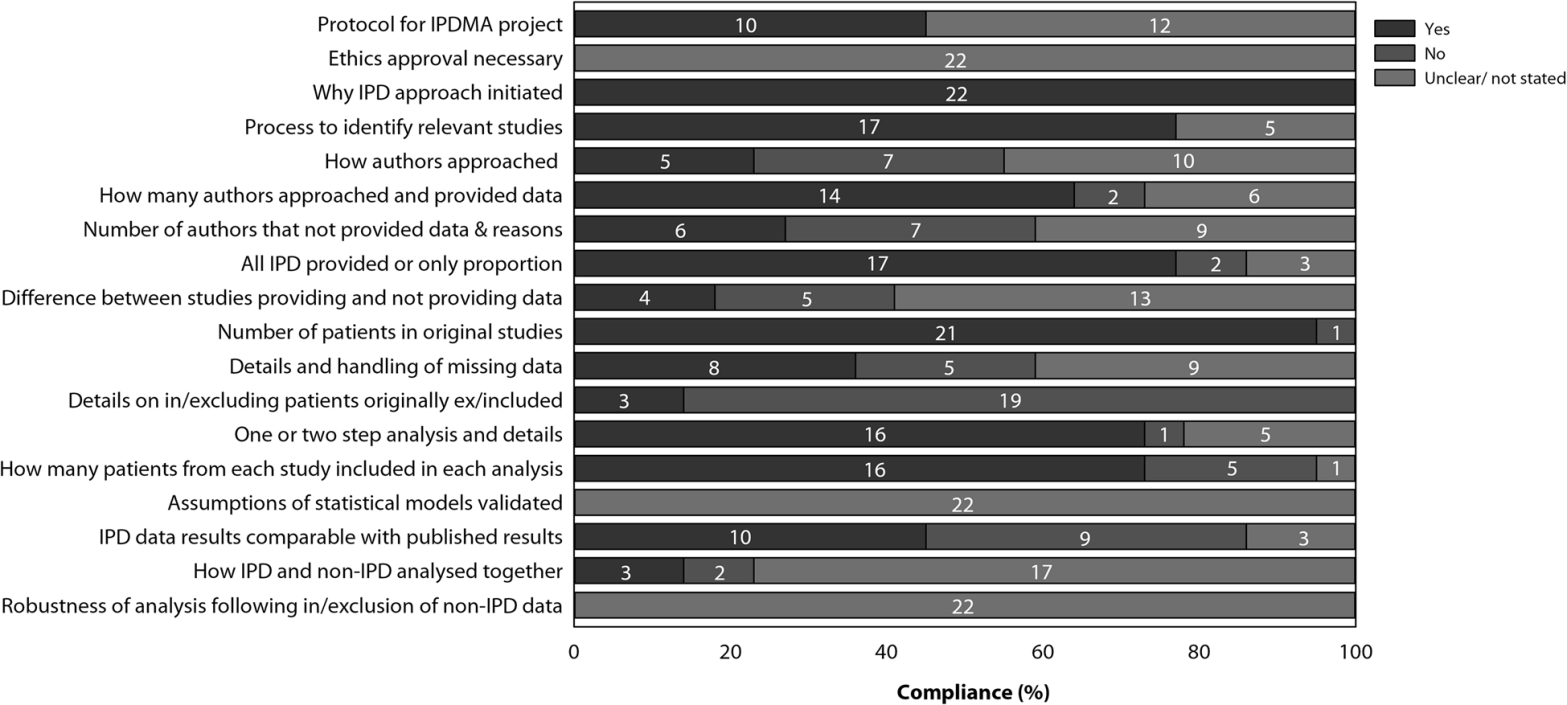
**Quality of IPDMA reporting surgical interventions.** Numbers inside bars are numbers of studies.

The quality of reporting of the surgical IPDMAs varied (Figure 3). More than half of the IPDMA failed to report whether or not there was a protocol for the IPDMA project available. The reason why the IPD approach was initiated and the numbers of patients within each of the original studies were generally well reported. For 17 (77%) IPDMAs, the process used to identify relevant studies for the IPDMA were reported. Details on the statistical analysis were reported in 16 (73%) IPDMAs, however, details on the identification process and statistical analysis were not described in one IPDMA (4%), and were unclear in the remaining five (23%) IPDMAs.

## Discussion

Our systematic review of all IPDMAs on surgical interventions published so far provides an overview of the potential advantages of IPDMAs (see Table 2 for examples). In 18 (82%) of the full set of 22 surgical IPDMAs assessed, subgroup analyses were performed to examine whether certain patients benefit more from a specific treatment than others. Eight (67%) of the 12 surgical IPDMAs with significant subgroups reported on what the implications of their findings were for clinical practice. Forty-four percent (8 out of 18) of the significant subgroups were implemented in clinical guidelines.

**Table 2:** Two examples of differences in conclusions with regard to how patient-level characteristics modify treatment effect

**Example**	**Description**
Effectiveness of coronary artery bypass grafting *vs*. percutaneous coronary interventions for multivessel disease.	A two-step meta-analysis of individual patient data from 7,812 patients included in 10 randomized trials comparing coronary artery bypass grafting (CABG) and percutaneous coronary intervention (PCI) in patients with multivessel coronary artery disease, showed a similar overall treatment effect on long-term mortality after CABG and PCI [20]. However, in diabetic patients mortality was substantially lower in the CABG group than in the PCI group (HR 0.70, 95% CI 0.56-0.87). Mortality was similar between groups in patients without diabetes (HR 0.98, 95% CI 0.86-1.12; *P*=0.01 for interaction). Patient age modified the effect of treatment on mortality with hazard ratios of 1.25 (95% CI 0.94-1.66) in patients aged <55 years, 0.90 (95% CI 0.75-1.09) in patients aged 55–64 years, and 0.82 (95% CI 0.70-0.97) in patients aged ≥65 years (*P*=0.002 for interaction). Treatment effect was not modified by other subgroups. CABG might be a better option for patients aged ≥65 years and patients with diabetes since mortality was found to be lower in these subgroups. These results have been implemented in clinical guidelines [41].
Effectiveness of routine *vs*. selective invasive strategy in patients with non-ST-segment elevation acute coronary syndrome.	An individual patient data meta-analysis of three randomized trials of routine *versus* selective invasive strategies in patients with non-ST-segment elevation acute coronary syndrome showed that a routine invasive strategy resulted in significantly less cardiovascular deaths (CV deaths) or non-fatal myocardial infarctions (MIs) compared to selective invasive strategies [30]. The authors used patient’s baseline characteristics to develop a multivariable risk prediction model. A simplified integer risk score was derived from the risk prediction model to predict a patient’s 5-year probability of CV death or MI, and the patients were categorized into three risk groups (low, intermediate, and high risk).
	The treatment effect was similar between groups in patients with low-risk (HR 0.80 (95% CI 0.63-1.02)) and intermediate-risk (HR 0.81 (95% CI 0.66-1.01)) scores. In patients with high-risk scores treatment favored routine over selective invasive strategies (HR 0.68 (95% CI 0.53-0.86)). There were 2.0% (95% CI −4.1-0.1%) and 3.8% (95% CI −7.4- -0.1%) absolute risk reductions in CV death or MI in the low- and intermediate-risk groups and an 11.1% (95% CI −18.4- -3.8%) absolute risk reduction in the highest-risk patients. The multivariable risk prediction model has not yet been implemented in clinical guidelines.

Although many IPDMAs performed subgroup analyses, the overall treatment was usually the main focus of the paper. Only occasionally subgroup analyses were emphasized. In surgical IPDMAs, similar to IPDMAs in general [42], subgroups were often based on patient and disease characteristics. The median number of subgroups has been reported to range from 2 to 4, the maximum number of subgroups from 15 to 50 [6,43-45], which is comparable to our findings. Justification of subgroup analyses, the methods used to perform subgroup analyses, and power calculations for performing subgroup analyses are often not reported in IPDMAs [6,43-48]. However, 11 (65%) of the IPDMAs included in our study justified at least one of the subgroups on which they reported, scientific literature being the mode of justification used. This is in line with other studies that found that clinical experience or biochemical justification is rare [44,46,49]. Others showed that the proportion of studies that used interaction tests for at least one of their subgroups ranges from 10% to 56% [6,43-48], which is slightly lower compared to our findings. So far, few studies mentioned the importance of the power of subgroup analyses [6,44,50,51], and reported that many reports put too much emphasis on subgroup analyses that commonly lacked statistical power. This is in agreement with the results of the present study.

To the best of our knowledge we are the first to study surgical IPDMAs, and illustrate the merits of this method within surgery. However, some potential limitations should also be discussed. First, our literature search for surgical IPDMAs was limited to IPDMAs with IPD obtained from RCTs comparing surgical interventions, excluding IPDMAs regarding drug-eluting medical devices and surgical trials in which a drug was the comparison, and records were limited to the English language. We, however, believe that our review provides a good representation of the method within the surgical field. Second, reporting bias could not be entirely excluded, since reporting of subgroup effects in scientific publications might be influenced by reviewers’ and editors’ opinions. Third, as most studies mentioned multiple subgroups, a clustering effect might occur for reporting on justification and statistical methods. Therefore, the results were reported on study level instead of individual subgroup level. Fourth, in the 12 IPDMAs of cardiovascular interventions reporting subgroup analyses, several studies included a same set of trials and the pattern of exploring heterogeneity among these studies might be similar, that is, there might be a clustering effect. As this might impact the subsequent analyses as well as the conclusion, we performed a sensitivity analysis. The outcomes and conclusions were not substantially influenced by the inclusion of studies using the same set of trials. Fifth, the time from publication to implementation of a result into a guideline or clinical practice can be highly variable, and sometimes takes even more than 10 years [52]. Most surgical IPDMAs included in the present study have only recently been published, and time to possible implementation has been rather short. Therefore, we might have underestimated the implementation of results from IPDMAs into guidelines and/or clinical practice.

## Conclusions

One of the challenges in medicine is to rationally implement available therapies in clinical practice, in the appropriate patients at the appropriate time. Findings from IPDMAs might provide insight into opportunities to improve evidence-based treatment decisions for patients. IPDMA is an extremely powerful tool if used correctly and provides the most definitive synthesis of the available evidence, also for potential subgroups. Despite the recommendations available on reporting clinical trials and meta-analyses, such as PRISMA [18], these guidelines have not been specifically developed for IPDMAs. The development of a generally accepted guideline for reporting on IPDMAs including subgroup analyses should therefore be encouraged. This seems the only option to really improve the reporting, analyses, and claims and applicability of subgroup effects in clinical research.

## Competing interests

The authors declare that they have no competing interests.

## Authors’ contributions

GH prepared the protocol with guidance from HG, CvL, and MR. GH and MR developed the search strategies. GH and MR selected relevant studies and extracted data; all authors participated in screening/extraction for the initial unpublished version of the review. GH carried out the analysis and prepared the manuscript with input from all authors. All authors read and approved the final manuscript.

## Supplementary Material

Additional file 1 Detailed search strategy. Click here for file

Additional file 2 PRISMA 2009 Checklist. Click here for file

Additional file 3 PRISMA 2009 Flow Diagram. Click here for file
