# Supplementary material for: A systematic review of individual patient data meta-analyses on surgical interventions
Source: Syst Rev. 2013 Jul 5;2:52. doi: 10.1186/2046-4053-2-52 (PMC3704956; doi:10.1186/2046-4053-2-52)
Supplement: Additional file 1 — Detailed search strategy. [file 2046-4053-2-52-S1.doc]

**Appendix A. Search strategy**

**Rationale**

Identify all IPDMAs of randomized controlled trials, aiming to identify the IPDMAs on surgical interventions. Initially, search of IPDMA was not restricted to particular treatments, disease outcome, or study types. Full text papers were retrieved when meta-analytic techniques for raw data (i.e., individual patient data) of randomized trials on surgical interventions were used.

**PubMed**

**Shojania & Berod [1] meta-analysis**

(((“meta-analysis”[pt] OR “meta-analysis”[tw] OR “metanalysis”[tw]) OR ((“review”[pt] OR “guideline”[pt] OR “consensus”[ti] OR “guideline*”[ti] OR “literature”[ti] OR “overview”[ti] OR “review”[ti]) AND ((“Cochrane”[tw] OR “Medline”[tw] OR “CINAHL”[tw] OR (“National”[tw] AND “Library”[tw])) OR (“handsearch*”[tw] OR “search*”[tw] OR “searching”[tw]) AND (“hand”[tw] OR “manual”[tw] OR “electronic”[tw] OR “bibliographi*”[tw] OR “database*”[tw] OR (“Cochrane”[tw] OR “Medline”[tw] OR “CINAHL”[tw] OR (“National”[tw] AND “Library”[tw]))))) OR ((“synthesis”[ti] OR “overview”[ti] OR “review”[ti] OR “survey”[ti]) AND (“systematic”[ti] OR “critical”[ti] OR “methodologic”[ti] OR “quantitative”[ti] OR “qualitative”[ti] OR “literature”[ti] OR “evidence”[ti] OR “evidence-based”[ti]))) NOT (“case*”[ti] OR “report”[ti] OR “editorial”[pt] OR “comment”[pt] OR “letter”[pt]))

AND

**Individual patient data**

("individual patient data"[All Fields] OR "individual patient"[All Fields] OR "patient data"[All Fields] OR "individual data"[All Fields] OR "individual patient data meta"[All Fields] OR "individual patient data meta analysis"[All Fields] OR "individual patient's data"[All Fields] OR "original patient data"[All Fields] OR "original data"[All Fields]) OR ("individual data analysis"[All Fields] OR "raw data"[All Fields] OR "raw patient data"[All Fields] OR "raw data analyses"[All Fields])

2,105

1. Shojania KG, Bero LA. Taking advantage of the explosion of systematic reviews: an efficient MEDLINE search strategy. Eff Clin Pract 2001;4(4):157-62.

**Embase**

‘meta-analysis’.mp AND ‘individual patient data’.mp

921

**Web of Science**

Topic=(“meta-analysis”) AND Title=(“individual patient data”)

522

**Cochrane Library (search restricted to reviews (no protocols))**

(meta-analysis):ti,ab,kw AND (individual patient data):ti,ab,kw

139
