# Supplementary material for: A systematic review of individual patient data meta-analyses on surgical interventions
Source: Syst Rev. 2013 Jul 5;2:52. doi: 10.1186/2046-4053-2-52 (PMC3704956; doi:10.1186/2046-4053-2-52)
Supplement: Additional file 3 — PRISMA 2009 Flow Diagram. [file 2046-4053-2-52-S3.doc]

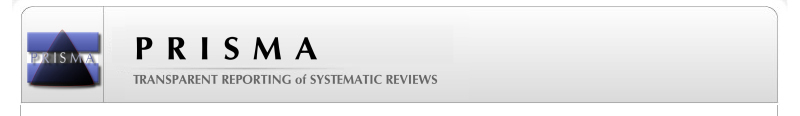
**PRISMA 2009 Flow Diagram**

**Screening**

**Included**

**Eligibility**

**Identification**

Records identified through database searching
(n = 3,597)

Additional records identified through other sources
(n = 0)

Records after duplicates removed
(n = 2,433)

Records screened
(n = 2,433)

Records excluded

(n = 2,391)

- Cohort, case report or case-control instead of RCT

- Methodological or tutorial review

- Objective differed too much from IPDMA

- Duplicate publications

- Conference abstracts & protocols

- Animal studies

- Non-surgical

Full-text articles assessed for eligibility
(n = 42)

Full-text articles excluded (n = 20)

- No IPDMA

- Duplicate publications

Studies included in qualitative synthesis
(n = 22)
